# Supplementary material for: Dietary Supplements for Female Infertility: A Critical Review of Their Composition
Source: Nutrients. 2021 Oct 11;13(10):3552. doi: 10.3390/nu13103552 (PMC8541636; doi:10.3390/nu13103552)
Supplement: Supplementary file 1 [file nutrients-13-03552-s001.zip › nutrients-1391258-supplementary.pdf]

nutrients

MDPI

Supplementary Materials

Table S1. List of dietary supplements (DS), their composition and score of expected efficacy. **S**: score of supplement's expected efficacy; **EV**: efficacy value of active ingredients in relation to literature and achievement of mED. **SOD**: super oxide dismutase. Ingredients without proven efficacy are **cursive underlined**. *DS containing at list one ingredient without evidence of efficacy.

**Active ingredients**	**DS 1**		**DS 2***		**DS 3**		**DS 4**		**DS 5**		**DS 6**		**DS 7***	
	**S = 7,50**		**S = 9,00**		**S = 7,50**		**S = 15,00**		**S = 7,50**		**S = 7,50**		**S = 9,00**	
	**Daily dose**	**EV**	**Daily dose**	**EV**	**Daily dose**	**EV**	**Daily Dose**	**EV**	**Daily dose**	**EV**	**Daily Dose**	**EV**	**Daily dose**	**EV**
Zinc														
Selenium														
Vitamin B12														
**Folic Acid**	200 µg	B	400 µg	A	400 µg	A	400 µg	A	400 µg	A	200 µg	B	400 µg	A
Glucomannan														
α-Lipoic Acid														
Pine bark extract														
**N-Acetyl Cysteine (NAC)**	600 mg	B												
**Coenzyme Q10**														
Gymnemic Acid														
Whey Protein														
Astaxanthin														
**Myo-inositol**	2000 mg	B	4000 mg	A	1100 mg	B	4000 mg	A	1100 mg	B	2000 mg	B	4000 mg	A
**Di-chiro-inositol**					27,6 mg	C			27,6 mg	C				
α-Tocopherol														
Vitamin C														
Alpha-lactoalbumin			100 mg	D									100 mg	D
**Melatonin**											1 mg	B		
Vitamin B1														
Vitamin B2														
Vitamin B6														
Betaine														
Resveratrol														
Manganese														
**Vitamin D3**														
SOD														
Chromium														
Glutathione														
Lutein														
Magnesium														
Bromelain														
Berberine														
Corosolic Acid														
Fucoxanthin														
Ginseng DE														
Cinnamon DE														
Silymarin														
Piperine														

Active ingredients	DS 8*		DS 9*		DS 10		DS 11*		DS 12*		DS 13*		DS 14*	
	S = 5,83		S = 3,33		S = 5,00		S = 2,50		S = 3,86		S = 0,63		S = 1,20	
	Daily dose	EV	Daily dose	EV	Daily dose	EV	Daily dose	EV	Daily dose	EV	Daily dose	EV	Daily dose	EV
Zinc					3 mg	D					10 mg	D		
Selenium											55 µg	D		
Vitamin B12													2,5 µg	D
**Folic Acid**	400 µg	A	200 µg	B	800 µg	A	400 µg	A	400 µg	A	400 µg	A	400 µg	A
Glucomannan														
α-Lipoic Acid	800 mg	D					600 mg	D	500 mg	D				
Pine bark extract														
**N-Acetyl Cysteine (NAC)**														
**Coenzyme Q10**														
Gymnemic Acid														
Whey Protein														
Astaxanthin														
**Myo-inositol**	2000 mg	B	200 mg	B					500 mg	B	2000 mg	B		
**Di-chiro-inositol**			500 mg	B	1200 mg	B	1000 mg	B						
α-Tocopherol											12 mg	D		
Vitamin C											160 mg	D		
Alpha-lactoalbumin														
**Melatonin**									1 mg	B				
Vitamin B1														
Vitamin B2														
Vitamin B6													1.4 mg	D
Betaine														
Resveratrol			24 mg	D									150 mg	D
Manganese			5 mg	D			10 mg	D	10 mg	D				
**Vitamin D3**			12,5 µg	C	20 µg	C			50 µg	C			25 µg	C
SOD							20 mg	D						
Chromium									200 µg	D				
Glutathione											50 mg	D		
Lutein											3 mg	D		
Magnesium														
Bromelain														
Berberine														
Corosolic Acid														
Fucoxanthin														
Ginseng DE														
Cinnamon DE														
Silymarin														
Piperine														

**Active ingredients**	**DS 15***		**DS 16***		**DS 17***		**DS 18***		**DS 19***		**DS 20***		**DS 21***	
	**S = - 0,60**		**S = 0,00**		**S = 0,63**		**S = 1,67**		**S = 0,50**		**S = 3,75**		**S = 3,33**	
	**Daily dose**	**EV**	**Daily dose**	**EV**	**Daily dose**	**EV**	**Daily dose**	**EV**	**Daily dose**	**EV**	**Daily dose**	**EV**	**Daily dose**	**EV**
Zinc	6 mg	D	10 mg	D										
Selenium	25 µg	D	55 µg	D										
Vitamin B12			2,5 µg	D	33 µg	D			10 µg	D	2,5 µg	D		
**Folic Acid**			400 µg	A	400 µg	A	400 µg	A			400 µg	A	200 µg	B
Glucomannan														
α-Lipoic Acid			200 mg	D	100 mg	D			150 mg	D				
Pine bark extract									100 mg	D				
**N-Acetyl Cysteine (NAC)**														
**Coenzyme Q10**			50 mg	C										
Gymnemic Acid							25 mg	D						
Whey Protein							37,5	D						
Astaxanthin			10 mg	D										
**Myo-inositol**	1500 mg	B	2000 mg	B	350 mg	B	2000 mg	B	2000 mg	B	1000 mg	B	1000 mg	B
**Di-chiro-inositol**									250 mg	C				
α-Tocopherol	5 mg	D	12 mg	D			12 mg	D	24 mg	D				
Vitamin C	300 mg	D	80 mg	D			80 mg	D	160 mg	D				
Alpha-lactoalbumin														
**Melatonin**														
Vitamin B1			1,1 mg	D	20 mg	D								
Vitamin B2			1,4 mg	D	20 mg	D								
Vitamin B6	0,6 mg	D			8 mg	D								
Betaine									100 mg	D				
Resveratrol	20 mg	D												
Manganese											2 mg	D	5 mg	D
**Vitamin D3**	5 µg	C	5 µg	C					10 µg	D				
SOD														
Chromium					200 µg	D								
Glutathione			50 mg	D										
Lutein														
Magnesium	200 mg	D												
Bromelain	40 mg	D												
Berberine														
Corosolic Acid														
Fucoxanthin														
Ginseng DE														
Cinnamon DE														
Silymarin														
Piperine														

**Active ingredients**	**DS 22***		**DS 23***		**DS 24***	
	**S = 7,50**		**S = 1,50**		**S = - 0,83**	
	**Daily dose**	**EV**	**Daily dose**	**EV**	**Daily dose**	**EV**
Zinc						
Selenium						
Vitamin B12						
**Folic Acid**	400 μg	A				
Glucomannan			4000 mg	D		
α-Lipoic Acid						
Pine bark extract						
**N-Acetyl Cysteine (NAC)**						
**Coenzyme Q10**						
Gymnemic Acid						
Whey Protein						
Astaxanthin						
**Myo-inositol**	2000 mg	B	1750 mg	B	100 mg	B
**Di-chiro-inositol**	400 mg	B	250 mg	C		
α-Tocopherol						
Vitamin C						
Alpha-lactoalbumin						
**Melatonin**						
Vitamin B1						
Vitamin B2						
Vitamin B6						
Betaine						
Resveratrol						
Manganese	10 mg	D				
**Vitamin D3**						
SOD						
Chromium					200 μg	D
Glutathione						
Lutein						
Magnesium						
Bromelain						
Berberine					400 mg	D
Corosolic Acid					2 mg	D
Fucoxanthin					20 mg	D
Ginseng DE					200 mg	D
Cinnamon DE					200 mg	D
Silymarin					80 mg	D
Piperine					9,5 mg	D
